# The ultrasound-guided nerve blocks of abdominal wall contributed to anesthetic management of cholecystectomy in a patient with Becker muscular dystrophy without using muscle relaxants

**DOI:** 10.1186/s40981-017-0134-1

**Published:** 2017-12-08

**Authors:** Masato Iwata, Naoya Kuzumoto, Yuka Akasaki, Masayo Morioka, Kana Nakayama, Nobuyoshi Matsuzawa, Katsuhiro Kimoto, Toshiyuki Shimomura

**Affiliations:** 1 Department of Anesthesiology, Nara Prefecture general Medical Center, 30-1, Hiramatsu 1-chome, Nara city, Nara 631-0846 Japan; 2 Department of Anesthesiology, Nara Medical University, Kashihara, Japan; 3 Perioperative Care Center, Nara Prefecture General Medical Center, Nara, Japan

**Keywords:** Becker muscular dystrophy, Total intravenous anesthesia, Rectus sheath block

## Abstract

Becker muscular dystrophy (BMD) is a progressive neuromuscular disorder caused by mutations in the dystrophin gene. The sensitivity to non-depolarizing muscle relaxant in a patient with muscle dystrophy is reportedly higher than that in normal individuals, and the duration of the effect is known to be prolonged. In this report, we present the case of a 58-year-old man with BMD who underwent laparoscopic cholecystectomy for symptomatic cholelithiasis under total intravenous anesthesia without the use of muscle-relaxant drugs and supplemented with regional anesthesia. Anesthesia was induced and maintained with propofol, remifentanil, and fentanyl; ultrasound-guided bilateral rectus sheath block (RSB) and right-sided subcostal transversus abdominis plane block (TAP) were performed. The procedure required conversion to open surgery because of hard conglutination; intraoperative and postoperative periods were uneventful. Adequate analgesia was maintained after extubation because of the effect of RSB and TAP.

## Background

Becker muscular dystrophy (BMD) is a relatively rare form of muscular dystrophy that has an incidence rate of approximately 1 in 18,000 live male births and a prevalence of 23.8 × 10^−6^ [1]. The condition results from mutations in the dystrophin gene [2] and is characterized by progressive weakness and wasting, predominantly of the proximal musculature. Patients with BMD undergoing surgery are susceptible to various intraoperative complications, such as aspiration pneumonia, prolonged artificial ventilation, malignant hyperthermia (MH), rhabdomyolysis, hyperkalemia, cardiomyopathy, hypotension, and arrhythmia [1, 3].

The use of non-depolarizing muscle relaxants in these patients is associated with an increase in the maximal effect and duration of action [1]. Owing to the risk of malignant hyperthermia and adverse reactions to volatile anesthetics, total intravenous anesthesia (TIVA) is preferred in these patients [1]. Rectus sheath block (RSB) is known to induce abdominal relaxation [4, 5]. In addition, RSB has been shown to reduce postoperative pain [5]. In our hospital, we perform RSB and right-sided subcostal transversus abdominis plane block to provide analgesia for laparoscopic cholecystectomy.

In this report, we present our experience with a case of BMD who underwent laparoscopic cholecystectomy under anesthesia with TIVA and RSB and without the use of muscle-relaxant drugs.

## Case presentation

A 58-year-old man (height 166 cm and weight 75 kg) was scheduled to undergo laparoscopic cholecystectomy for symptomatic cholelithiasis. He was diagnosed with BMD at 12 years. He had difficulty in walking because of weakness in leg muscles. Pulmonary function tests revealed a forced vital capacity of 50% of the predicted levels and forced expiratory volume in 1 s of 92% of predicted levels. He had not experienced any episode of cardiac failure; his electrocardiogram was almost normal.

General anesthesia was induced with the effect site concentration at 4 μg/mL propofol using a target-controlled infusion (TCI) pump (Terufusion TE-371, Terumo, Tokyo, Japan), 0.2 mg fentanyl, and 0.25 μ/kg^/^min remifentanil. Rocuronium was not used. He was maintained on 2.5–3 μg/mL propofol with TCI and 0.1–0.15 μg/kg^/^min remifentanil with oxygen–air mixture. Anesthesia was maintained under TIVA without the use of muscle relaxants. After induction, images of the rectus abdominis muscle were monitored with LOGIQ e^®^(GE healthcare, Tokyo, Japan). Using a 20-G Tuohy needle (80 mm Hakko medical, Nagano, Japan), we performed ultrasound-guided bilateral RSB and right-sided subcostal TAPB with 0.75% ropivacaine 20 mL and 1% lidocaine 20 mL, for abdominal relaxation.

Mid-way through the surgery, the procedure required conversion to open surgery because of hard conglutination; however, non-depolarizing muscle relaxants were not required. As an analgesic, 1000 mg of acetaminophen was administered. After the completion of surgery, he was extubated and the patient did not feel any pain because of the wound.

For pain relief, flurbiprofen axetil 50 mg div was medicated after he was transferred to the surgical ward, after 8, and 16 h from the operation. Numerical Rating Scale regarding the wound pain changed 4~6 on the surgery day and 2~3 on postoperative day 1. Postoperative complication of bile leak afflicted him, but on postoperative day 93, the patient was discharged.

## Discussion

We reported that bilateral rectus sheath block and right-sided subcostal transversus abdominis plane block contributed to successful anesthetic management of laparoscopic cholecystectomy, conversed to open cholecystectomy under total intravenous anesthesia without the use of muscle relaxants, in a patient with BMD. Because of the involvement of complex multisystems in BMD patients undergoing surgery, several specific perioperative considerations may significantly impact on surgical outcomes [1, 2]. Several reports on the management of anesthesia in BMD patients are available in the published literature [1–3]. Up to 50–70% patients with muscular dystrophy manifest cardiac abnormalities, although it is clinically significant in only 10% of these patients [6]. An estimated 30% of deaths in individuals with Duchenne muscular dystrophy (DMD) are due to respiratory causes. Therefore, careful preoperative pulmonary assessment is of critical importance. Reports have suggested a relationship between DMD or BMD and MH. Use of volatile anesthetics in patients with muscular dystrophy is associated with an increased risk of rhabdomyolysis, which could result in cardiac arrest and MH [3]. Therefore, volatile anesthetic agents should be avoided and TIVA is recommended in these patients [7].

Limited use of non-depolarizing muscle relaxants may be allowed depending on situations in consideration of increased maximal effect and duration of action [8]. However, a literature is available that reports the safe use of sugammadex to antagonize the neuromuscular block and rapid recovery in DMD [9].

## Conclusions

In this report, we present a case of BMD in whom laparoscopic cholecystectomy was performed under TIVA and RSB and without the use of muscle-relaxant drugs; good postoperative analgesia was achieved with this regime.
